# Assessment of Anti-Inflammatory and Antioxidant Activities of a Proprietary Preparation of Quercetin–Rutin Blend (SophorOx™) in Exercised Rats

**DOI:** 10.1155/2024/9063936

**Published:** 2024-02-10

**Authors:** Devanand Shanmugasundaram, James Martin Roza

**Affiliations:** ^1^Compliance and Operations (Toxicology), Vedic Lifesciences Pvt. Ltd., Mumbai, India; ^2^Layn Natural Ingredients, 36 Executive Park, Irvine 250, CA 92614, USA

## Abstract

**Objectives:**

Flavonoids comprise a huge class of phenolic compounds widely distributed throughout the plant kingdom. Although quercetin and rutin have been studied individually for their therapeutic value, the synergistic effect of combining the two has previously not been measured. The objective of this trial was to evaluate the anti-inflammatory and antioxidant properties of both quercetin and rutin when combined in the form of SophorOx™ (a proprietary preparation of quercetin–rutin) in exercised rats.

**Methods:**

Sprague–Dawley rats were orally administered SophorOx™ at 500 mg·kg^−1^·b.w. and subjected to daily exercise on a fabricated treadmill for 4 weeks. A total of 24 animals were randomly divided into four groups. All the animals were examined for body weight, feed consumption, signs of clinical abnormalities, and morbidity. In addition, serum collected on days 8, 15, 22, and 29 were measured for the liver function test (LFT), random blood sugar (RBS), inflammatory markers C-reactive protein (CRP), oxidative stress markers (8-isoprostane (8-iso-PGF2*α*), malondialdehyde (MDA), 8-hydroxydeoxyguanosine (8-OHdG), and cytokine levels interleukin-1*β* (IL-1*β*), interleukin 6 (IL-6), and tumor necrosis factor-*α* (TNF-*α*)) by the ELISA method.

**Results:**

Rats that received SophorOx™ showed no signs of adverse effects, and no significant changes were observed in body weight, feed consumption, liver enzymes, and blood glucose levels. The exercise-treated rats administered with SophorOx™ exhibited a significant reduction in oxidative and inflammatory marker levels, *viz*., CRP (113.32 ng·mL^−1^) and oxidative stress markers 8-OHdG (19.32 pg·mL^−1^), MDA (1.06 nmol·mL^−1^), 8-iso-PGF2*α* (1.29 ng·mL^−1^), IL-1*β* (0.77 pg·mL^−1^), and IL-6 (317.14 pg·mL^−1^) in comparison to those rodents that were exercised without SophorOx™.

**Conclusion:**

Oral administration of SophorOx™ significantly reduced oxidative stress and inflammatory marker levels when measured in the rodents subjected to high-intensity exercise.

## 1. Introduction

The human body's antioxidant defence system plays a crucial role in preventing or delaying the oxidation of extracellular and intracellular biomolecules [1, 2]. However, the level of antioxidants in the body can be altered by physiological stimuli, *viz*., smoking, radiation, alcohol use, and exercise, which can increase the oxidative stress the body experiences under those conditions [3, 4]. Free radicals and reactive oxygen species (ROS) levels are amplified when oxidative stress occurs. As a result, they can cause oxidative damage to DNA and initiate an inflammatory response due to muscle damage, allowing the infiltration of inflammatory mediators [5, 6]. These inflammatory mediators, viz., prostaglandin E2 (PGE2), tumor necrosis factor-*α* (TNF-*α*), cyclooxygenase-2 (COX-2), and interleukin-1*β* (IL-1 *β*), trigger macrophages to synthesize C-reactive protein (CRP), which in turn activates the creation of additional free radicals in the body [4].

The source of antioxidants can be natural or artificial. Flavonoids are compounds rich in antioxidant activity that helps the body ward off routine toxins. Flavonoids are one of the most important groups of polyphenolic compounds. These bioactive constituents are commonly found in nature, and their presence is ubiquitous [7]. They are widely consumed in foodstuffs [8] for their antioxidant, anti-inflammatory, anticarcinogenic, anticoagulation, anti-ischemic, and neuroprotective properties [9, 10]. Quercetin and rutin are some of the most widely distributed dietary flavonoids and exist in high concentrations in certain plants. Quercetin and its metabolites possess antioxidant, anticarcinogenic, and anti-inflammatory activities [11]. Rutin, a quercetin glycoside, is extensively found in many plants and recognised for its antioxidant, antiarthritic, neuroprotective, and anti-inflammatory properties [12].

Though numerous studies are being documented on quercetin and rutin, this will be the first of its kind to measure the combined beneficial effects of quercetin and rutin preparation on a stress-induced rat model. Before this *in vivo* trial, we screened SophorOx™ in LPS-stimulated RAW 264.7 macrophages for its potent antioxidant and anti-inflammatory activities [13]. The combination of quercetin–rutin significantly reduced LPS-induced production of TNF-*α* and IL-6 (∼30% inhibition). In contrast, the generation of LPS-stimulated NO/nitrite and ROS levels was significantly reduced at low concentrations by a quercetin–rutin (<3 *μ*M) treatment. In the present study, we aimed to evaluate the antioxidant and anti-inflammatory effects of an oral administration of quercetin–rutin on stress-induced biomarkers. High-intensity exercise induces reactive oxygen species (ROS) formation and activates inflammatory cascades. Therefore, treadmill exercise provides a useful platform for studying the effectiveness of a polyphenolic extract in mitigating ROS and inflammation through the use of biomarkers widely accepted in the scientific community. The biomarkers used in this study are commensurate with the objective of this research in determining the effect of a polyphenolic extract (SophorOx) in deducing oxidative stress and inflammation. 80HdG is one of the most predominant forms of free radical-induced oxidation. Along with 8-isoprostane, MDA, IL-6, and CRP, all reliable biomarkers, the results of this study provided a comprehensive assessment of SophorOx's effects on oxidation and inflammation. We hypothesized that oral administration of quercetin–rutin (SophorOx™) (500 mg·kg^−1^) [14–17] would be associated with the modulation of the immune system by inhibiting oxidative stress and reducing the secretion of proinflammatory cytokines.

## 2. Materials and Methods

### 2.1. Chemicals, Reagents, and Kits

Kits for 8-iso-PGF2*α* ELISA (Lot. No. R8ISP0321), Rat CRP/PTX1 ELISA (Lot. No. RCRPTX10321), Rat MDA ELISA (Lot. No. RMDA0321), Rat 8-OHdG ELISA (Lot. No. R8OHDG0321), GenLISA™ Rat TNF-*α* ELISA (Lot. No. K02-0063), Rat Interleukin-1*β* ELISA (Lot. No. RIL1B0321), and GenLISA™ Rat IL-6 ELISA (Lot. No. RI60321) were procured from Krishgen BioSystems, India. Glucometer strips (Lot No. C038130) were purchased from SD Biosensor Healthcare Pvt. Ltd., India. All the chemicals and reagents used for the trial were of analytical grade.

### 2.2. SophorOx™ Preparation

The quercetin–rutin blend (proprietary preparation of SophorOx™) was prepared from the buds of *Sophora japonica* L. by following the standard operating procedures. The buds are cleaned and milled and then underwent ethanol/water extraction to capture the polyphenolic components inherent to the plant. These components are further centrifuged, crystallized, concentrated, and recrystallized to arrive at their final state, which is composed of 50.67% quercetin, 41.26% rutin, and ∼2% other flavonoids (i.e., kaempferol (0.88%), isorhamnetin (1.33%), genistin (0.14%), and genistein (0.005%)).

The extract was additionally concentrated and dried to get a free-flowing crystalline powder with an active content of quercetin: 50.67%, and rutin: 41.26% (Batch No. QUE06-20103001), a bright yellow crystalline powder with a manufacturing date: 30^th^ October 2020 and expiry date: 29^th^ October 2023, which was stored at room temperature in a tightly sealed container that was moisture free and protected from strong light. SophorOx™ was then subjected to all quality screening procedures and microbial residue analysis and sealed for experimental purposes. Studies assessing the safety and effectiveness have shown no serious adverse effects at up to 2,000 mg/day [18–20]. Dietary supplement usage levels typically range from 500 to 1,000 mg daily. Based on this, SophorOx™ was administered orally at a dose of 500 mg·kg^−1^·b.w. till the end of the experimental protocol. The test material was prepared before each dosing based on the individual body weights of the animals.

### 2.3. Experimental Animals and Welfare

The experimental protocol was conducted by following the Committee for the Purpose of Control and Supervision of Experiments on Animals (CPCSEA) and Institutional Animal Ethics Committee (IAEC) guidelines with standard operating procedures (approval no. VIP/IAEC/235/2021). Adult male Sprague–Dawley rats (∼8 weeks old, mean body weight of ∼230–240 g) were employed for the study. Animals were procured from a CPCSEA-approved vendor lab, Hylasco Bio-Technology Pvt. Ltd., India, in compliance with the ethical practices laid down in the guidelines for animal care and accredited by the American Association for Accreditation of Laboratory Animal Care (AAALAC), USA. The animals were subjected to a veterinary examination and allowed to acclimatize to the laboratory environment for five days. Animals were provided access to feed and water *ad libitum* as per experimental conditions (temperature 23 ± 2°C; relative humidity 50 ± 10%; and 12 h alternate light/dark cycle with 12–15 cycles/hour of air change). Reverse osmosis water and commercial pellet feed were provided *ad libitum* for 4 weeks.

### 2.4. Experimental Design

The study was conducted using 24 adult male SD rats that were randomly divided into four groups (*n* = 6):

- Group 1 (G1): animals were exercised on a treadmill, and no treatment was given
- Group 2 (G2): animals received SophorOx™ through oral administration (500 mg·kg^−1^·b.w) for 4 weeks without exposure to exercise
- Group 3 (G3): animals were exercised and received SophorOx™ orally (500 mg·kg^−1^·b.w) for 4 weeks
- Group 4 (G4): standard control, where the animals were not exercised, and no SophorOx™ was administered.

### 2.5. Forced Treadmill Exercise

Animals of respective groups were subjected to daily running on a fabricated treadmill (PowerMax). The treadmill's running platform was fabricated using a special lane box of Perspex and reformed into a 3-lane rodent treadmill that allowed three animals to run simultaneously. The specified group's animals were exercised daily over a treatment period of 4 weeks. The rats were initially allowed to explore the treadmill freely and exercise at a speed of 6.0 m/minute to get acclimatized. Subsequently, the rate increased to 30 meters/minute during the first and second day of week 1 and then elevated to 40 meters/minute by the 4^th^ day of that week. Finally, animals were exercised once daily (frequency) for 45 meters/minute (time duration) till the remainder of the treatment period. The treadmill exercise was established at the test facility after several validations and the standard operating procedures described in the study design.

### 2.6. In-Life Observations

The animals were monitored weekly for body weight gain and feeding patterns. In addition, any clinical signs of toxicity, including changes in the skin, fur, eyes, and mucous membranes, the occurrence of secretions and excretions, and autonomic activity (lacrimation, piloerection, pupil size, and respiratory pattern) were recorded daily during the treatment period. Similarly, any changes in gait, posture, and response to handling and the presence of clonic or tonic movements, stereotypes (repetitive circling), or bizarre behaviour were also recorded [21]. The blood samples were collected from each animal on the study's 8^th^, 15^th^, 22^nd^, and 29^th^ days. Samples were collected in the morning before treatment, and blood was collected through the tail vein using a 5 mL syringe with a 23 G needle. All the animals were euthanized on terminal sacrifice using isoflurane (20% v/v in propylene glycol in a glass vacuum desiccator).

### 2.7. Biochemical Parameters and Biomarkers

Blood samples for glucose estimation were collected in a sodium fluoride vial from each animal and measured on the first day of every week before exercise and treatment doses using an SD CodeFree™ Glucometer. In addition, the blood samples for LFT were collected and allowed to stand for complete clotting and further centrifuged at 10,000 rpm for 5 minutes at 4°C. The collected serum was separated and stored at −80°C for the following parameters, *viz*., aspartate aminotransferase (AST), alanine aminotransferase (ALT), alkaline phosphatase (ALP), and total bilirubin (T-BIL) using the fully automated clinical chemistry analyzer Rx Daytona+ by Randox Laboratories, UK. All the inflammatory markers, C-reactive protein (CRP), and oxidative stress markers (8-isoprostane (8-iso-PGF2*α*), malondialdehyde (MDA), 8-hydroxydeoxyguanosine (8-OHdG) and cytokine levels interleukin-1*β* (IL-1*β*), interleukin 6 (IL-6), and tumor necrosis factor *α* (TNF-*α*)) were determined by the enzyme-linked immunoassay (ELISA) method as per the Krishgen BioSystems protocol.

### 2.8. Statistical Analysis

Data were expressed as the mean ± standard deviation (SD). Statistical analysis was performed using one-way analysis (ANOVA) and GraphPad Prism software (Version 7.04, San Diego, CA, USA), followed by Dunnett's test for multiple comparisons to determine the difference in efficacy among various groups. The value for *p* < 0.05 was considered statistically significant.

## 3. Results

### 3.1. Clinical Signs and Mortality

Oral administration of SophorOx™ did not show any incidence of mortality or abnormal clinical signs among the treated groups. Furthermore, no treatment-related abnormalities in home cage observations, *viz*., changes in the skin, fur, eyes, and mucous membranes, the occurrence of secretions and excretions, or autonomic activity (lacrimation, piloerection, pupil size, and respiratory pattern), were observed. In addition, there were no changes in gait, posture, and response to handling nor were there clonic or tonic movements, repetitive circling, or bizarre behaviour observed in any of the groups.

### 3.2. Feed Intake and Bodyweight

The feed and water intake patterns did not vary among all four groups. The mean feed consumption was observed as ∼21 g/day/animal, which was statistically insignificant compared to the control group. Bodyweight was measured throughout the treatment period; no statistically significant difference was observed in any group. The terminal body weight of animals was in the range of 280–290 g across the groups.

### 3.3. Biochemical Parameters

**Figure 1:**
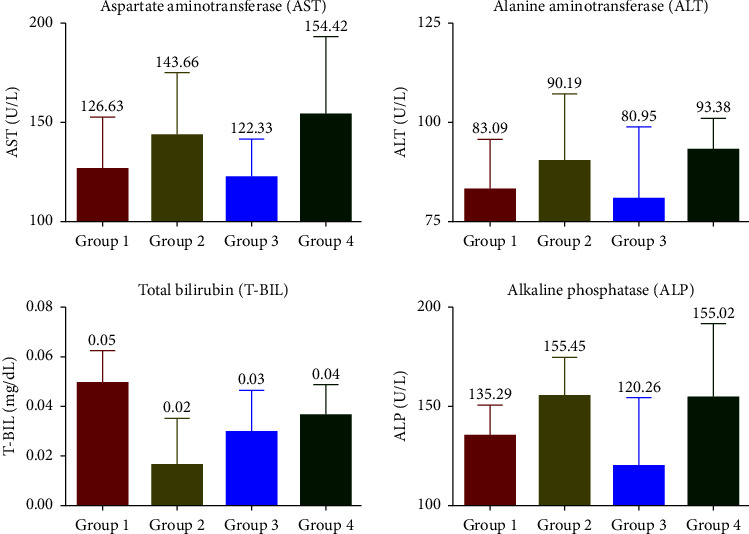
Effect of SophorOx™ on the liver function test (LFT). Group 1: animals were exercised and no treatment; group 2: animals received SophorOx™ (500 mg·kg^−1^·b.w); group 3: animals were exercised and received SophorOx™ orally (500 mg·kg^−1^·b.w). Group 4 (G4): standard control was not exercised and no SophorOx™ was administered. All results were expressed as mean ± SD (*n* = 6). Data analysis (GraphPad Prism Software Version 7.04, San Diego, CA, USA) was performed using one-way ANOVA followed by Dunnett's test for multiple comparisons. No statistically significant change was observed across the groups.

No significant change in blood glucose levels was found in SophorOx™-treated groups compared to the control group (G4). The blood glucose levels were within normal reference ranges (123 ± 38 mg/dL) during treatment. In the liver function test, the measured values of all parameters (AST, ALT, T-BIL, and ALP) in the treatment groups were statistically insignificant within the reported ranges. Hence, the treatment given to the rats did not alter their liver function, as demonstrated in Figure 1.

### 3.4. Effects of SophorOx™ on Oxidative Stress Markers

**Figure 2:**
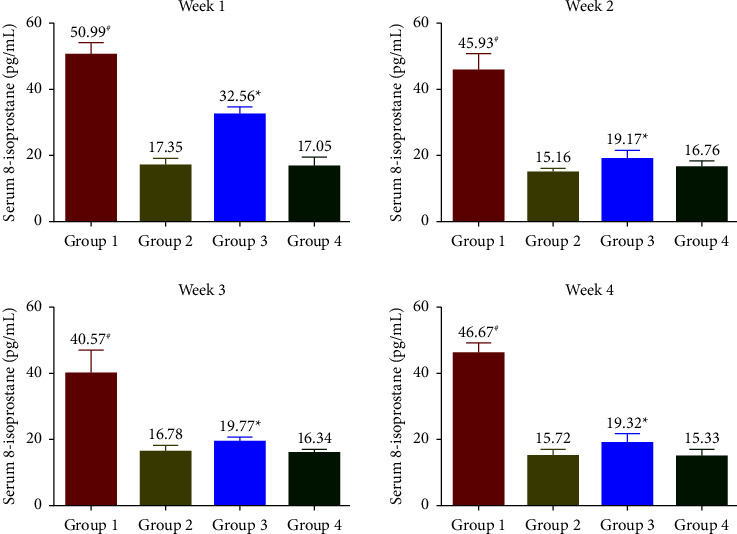
Effect of SophorOx^TM^ on serum 8-isoprostane levels. Group 1: animals were exercised and no treatment; group 2: animals received SophorOx™ (500 mg·kg^−1^·b.w); group 3: animals were exercised and received SophorOx™ orally (500 mg·kg^−1^·b.w). Group 4 (G4): standard control was not exercised and no SophorOx™ was administered. All results were expressed as mean ± SD (*n* = 6). Data analysis (GraphPad Prism Software Version 7.04, San Diego, CA, USA) was performed using one-way ANOVA followed by Dunnett's test for multiple comparisons. 8-Iso-PGF2*α* levels of the G3 groups showed significant changes from week 1 to the end of the treatment period. ^*∗*^Statistically significant (*p* < 0.05) in comparison with G4.

**Figure 3:**
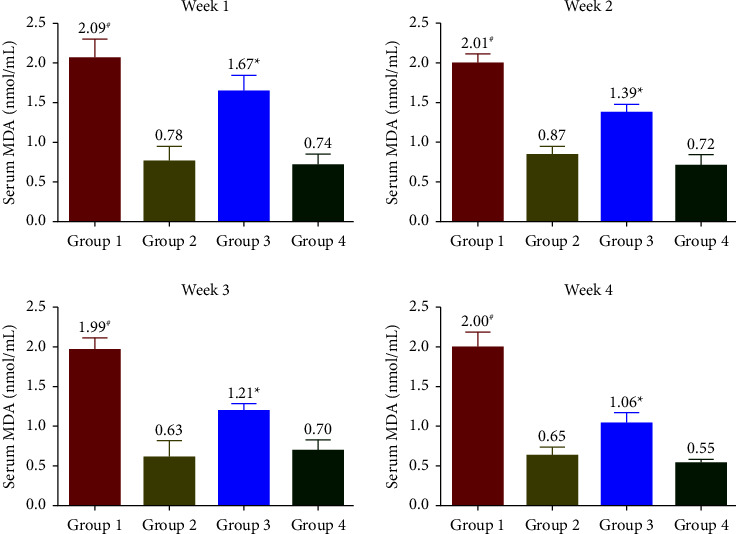
Effect of SophorOx^TM^ on serum MDA levels. Group 1: animals were exercised and no treatment; group 2: animals received SophorOx™ (500 mg·kg^−1^·b.w); group 3: animals were exercised and received SophorOx™ orally (500 mg·kg^−1^·b.w). Group 4 (G4): standard control was not exercised and no SophorOx™ was administered. All results were expressed as mean ± SD (*n* = 6). Data analysis (GraphPad Prism Software Version 7.04, San Diego, CA, USA) was performed using one-way ANOVA followed by Dunnett's test for multiple comparisons. MDA levels of the G3 group showed significant changes from week 1 to the end of the treatment period. ^*∗*^Statistically significant (*p* < 0.05) in comparison with G4.

**Figure 4:**
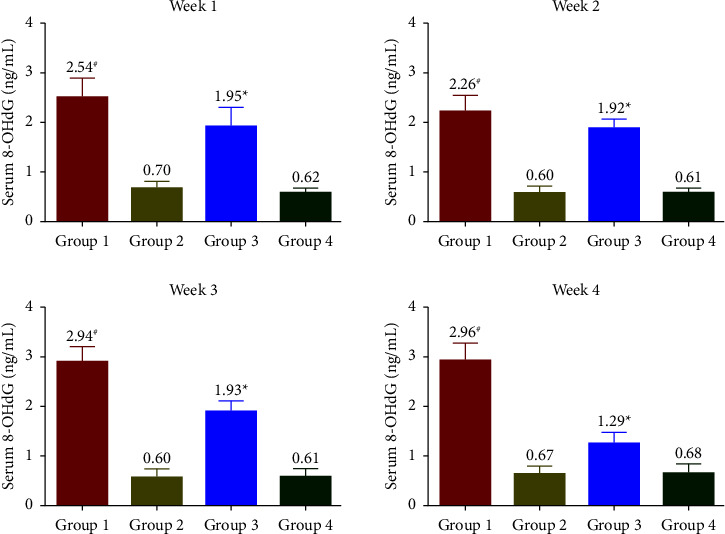
Effect of SophorOx^TM^ on serum 8-OHdG levels. Group 1: animals were exercised and no treatment; group 2: animals received SophorOx™ (500 mg·kg^−1^·b.w); group 3: animals were exercised and received SophorOx™ orally (500 mg·kg^−1^·b.w). Group 4 (G4): standard control was not exercised and no SophorOx™ was administered. All results were expressed as mean ± SD (*n* = 6). Data analysis (GraphPad Prism Software Version 7.04, San Diego, CA, USA) was performed using one-way ANOVA followed by Dunnett's test for multiple comparisons. 8-OHdG levels of the G3 groups showed significant changes from week 1 to the end of the treatment period. ^*∗*^Statistically significant (*p* < 0.05) in comparison with G4.

The levels of 8-iso-PGF2*α* (Figure 2), MDA (Figure 3), and 8-OHdG (Figure 4) were measured to be increased throughout the treatment period in G1. At the same time, G2 had similar levels of stress markers equivalent to G4. In G3, a significant reduction in levels of oxidative biomarkers was observed in the 1^st^ week. In contrast, a more pronounced reduction was observed from the 2^nd^ week onwards (*p* < 0.05) and reached similar levels comparable to G4 at the end of the treatment phase. All oxidative stress biomarkers (8-iso-PGF2*α*, MDA, and 8-OHdG) of the G1 group showed increased levels compared with G3 during the experimental period.

### 3.5. Effects of SophorOx™ on Inflammatory Biomarkers

**Figure 5:**
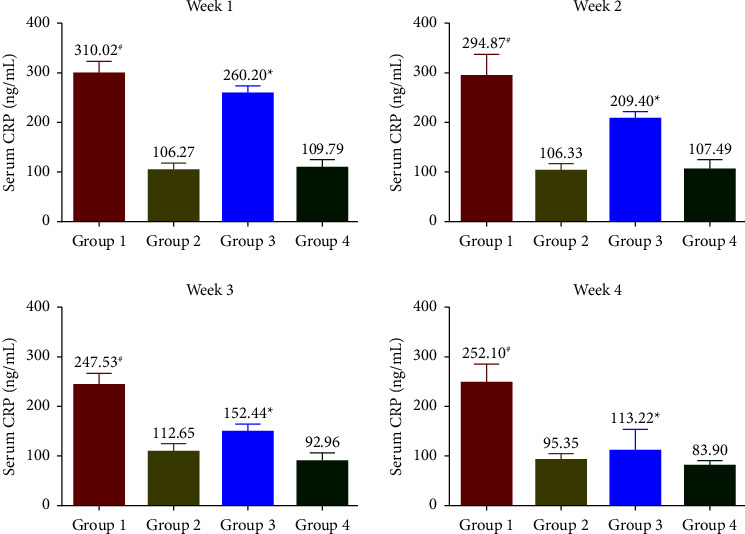
Effect of SophorOx^TM^ on serum CRP levels. Group 1: animals were exercised and no treatment; group 2: animals received SophorOx™ (500 mg·kg^−1^·b.w); group 3: animals were exercised and received SophorOx™ orally (500 mg·kg^−1^·b.w). Group 4 (G4): standard control was not exercised and no SophorOx™ was administered. All results were expressed as mean ± SD (*n* = 6). Data analysis (GraphPad Prism Software Version 7.04, San Diego, CA, USA) was performed using one-way ANOVA followed by Dunnett's test for multiple comparisons. CRP levels of the G3 groups showed significant changes from week 1 to the end of the treatment period. ^*∗*^Statistically significant (*p* < 0.05) in comparison with G4.

**Figure 6:**
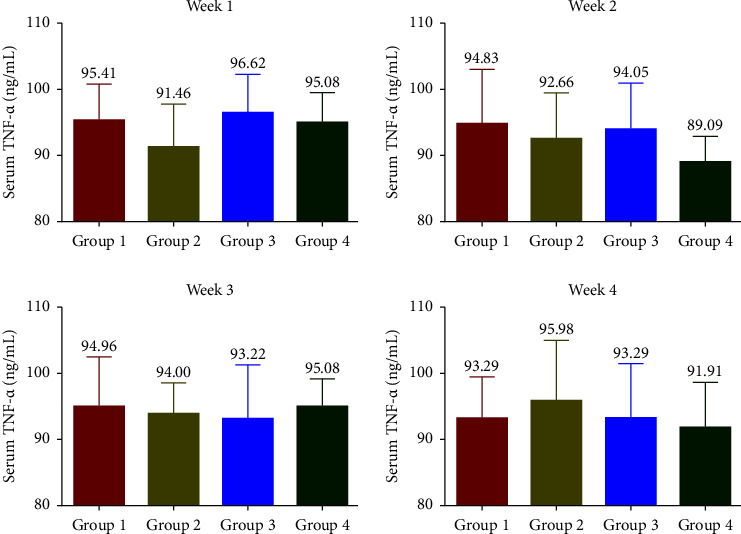
Effect of SophorOx^TM^ on serum TNF-*α* levels. Group 1: animals were exercised and no treatment; group 2: animals received SophorOx™ (500 mg·kg^−1^·b.w); group 3: animals were exercised and received SophorOx™ orally (500 mg·kg^−1^·b.w). Group 4 (G4): standard control was not exercised and no SophorOx™ was administered. All results were expressed as mean ± SD (*n* = 6). Data analysis (GraphPad Prism Software Version 7.04, San Diego, CA, USA) was performed using one-way ANOVA followed by Dunnett's test for multiple comparisons. No statistically significant change was observed across the groups.

**Figure 7:**
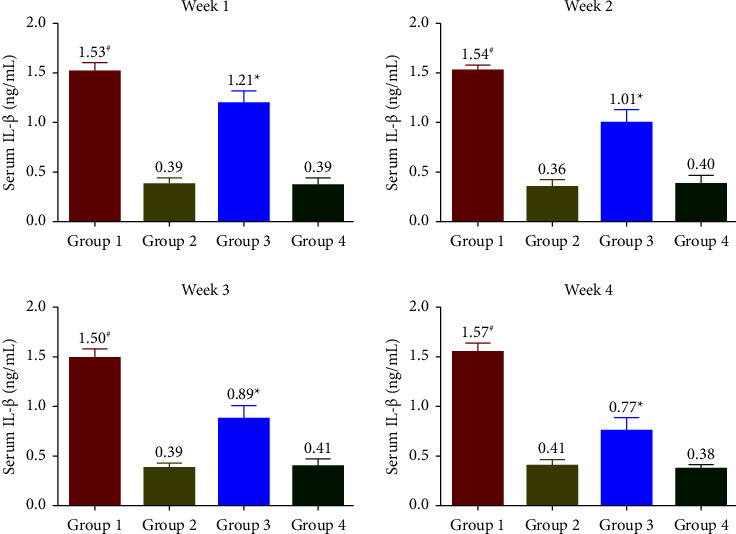
Effect of SophorOx™ on serum IL-1*β* levels. Group 1: animals were exercised and no treatment; group 2: animals received SophorOx™ (500 mg·kg^−1^·b.w); group 3: animals were exercised and received SophorOx™ orally (500 mg·kg^−1^·b.w). Group 4 (G4): standard control was not exercised and no SophorOx™ was administered. All results were expressed as mean ± SD (*n* = 6). Data analysis (GraphPad Prism Software Version 7.04, San Diego, CA, USA) was performed using one-way ANOVA followed by Dunnett's test for multiple comparisons. IL-1*β* levels of G3 groups showed a significant reduction from week 1 to the end of the treatment period (approximately 50% reduction). ^*∗*^Statistically significant (*p* < 0.05) in comparison with G4.

**Figure 8:**
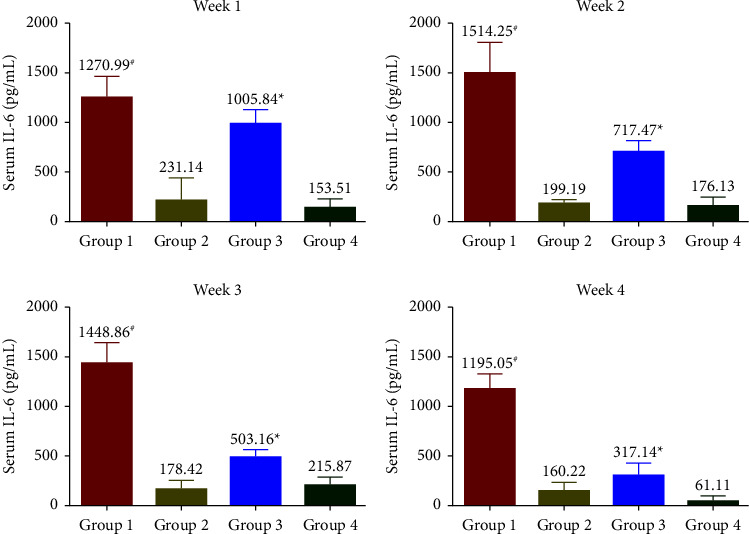
Effect of SophorOx^TM^ on serum IL-6 levels. Group 1: animals were exercised and no treatment; group 2: animals received SophorOx™ (500 mg·kg^−1^·b.w); group 3: animals were exercised and received SophorOx™ orally (500 mg·kg^−1^·b.w); group 4 (G4): standard control was not exercised and no SophorOx™ was administered. All results were expressed as mean ± SD (*n* = 6). Data analysis (GraphPad Prism Software Version 7.04, San Diego, CA, USA) was performed using one-way ANOVA followed by Dunnett's test for multiple comparisons. IL-6 levels of the G3 groups showed a significant reduction from week 1 to the end of the treatment period (approximately 25% reduction). ^*∗*^Statistically significant (*p* < 0.05) in comparison with G4.

The levels of CRP (Figure 5) were measured to be increased throughout the treatment period in G1. Treatment with SophorOx™ alone (G2) had similar CRP levels to G4. In G3, where the animals received both exercise and SophorOx™, a significant reduction in CRP levels was observed in the 1^st^ week. Furthermore, the CRP marker levels reduced steadily over the treatment period of 3 weeks, and the results measured were similar to those in G4. TNF-*α* levels (Figure 6) remained unchanged across all the groups during the experimental period. The levels of IL-1*β* and IL-6 were found to be highest in G1 throughout the study period. The IL-1*β* (Figure 7) and IL-6 (Figure 8) levels of G2 were almost similar to those in G4. However, G3 significantly reduced IL-1*β* and IL-6 levels in the 1^st^ week. By the end of treatment, the results for G3 showed a remarkable reduction (statistical significance) in IL-1*β* levels of 50% and IL-6 of 25% compared to G4. CRP, IL-1*β*, and IL-6 of the G1 group showed increased levels compared with G3 during the experimental period.

## 4. Discussion

The levels of oxygen uptake are increased dramatically in various organs during strenuous physical activity, especially in the skeletal muscles. The repetitive muscle contraction increases oxygen levels and accumulates reactive oxygen species (ROS). Therefore, free radicals and reactive oxygen species (ROS) production are augmented by exhaustive exercises and other stresses [22, 23]. Flavonoids are drawing attention among naturally found bioactive compounds due to their wide-ranging therapeutic properties [24]. Quercetin and rutin mediate anti-inflammatory responses by inhibiting the expression of COX1 and COX2 enzymes [25]. Similarly, rutin also possesses various health-promoting properties as those attributed to quercetin [26].

The animals tolerated the daily exercise procedure very well, and none of the exercised animals displayed any signs of discomfort or detrimental response during the entire experimental period. All groups showed comparable body weights, and a similar trend in body weight gain was seen in all groups throughout the study's duration. A similar study conducted by Groussard et al. [27] also demonstrated comparable weight gain in all treatment groups of the stressed rat models.

Blood glucose levels remained unaltered throughout the study. Quercetin is believed to interact with many molecular targets in the skeletal muscle, small intestine, adipose tissue, liver, and pancreas to control whole-body glucose homeostasis [28]. LFT parameters (AST, ALT, T-BIL, and ALP) were also measured within a normal range across all groups, demonstrating that the administration of SophorOx™ did not adversely impact liver function.

The oxidative biomarkers (8-iso-PGF2*α* and MDA) and 8-OHdG were assessed to evaluate stress-induced rats' physical and physiological status. In the SophorOx™ and exercised group (G3), the levels of biomarkers were significantly reduced in the 1^st^ week and throughout the terminal phase of the experiment. This might be due to the combination of quercetin–rutin and its defence mechanism in stress-induced oxidative pathways. C-reactive protein (CRP) levels are generally elevated postmacrophage activation as an inflammatory response to stress. In this study, it has been reported that the oral administration of SophorOx™ resulted in a significant reduction of CRP levels in the 1^st^ week (*p* < 0.05) and further reached basal levels by the end of the trial.

The release of cytokines further mediates the inflammatory processes. In addition, they transduce the signals between cells to elicit an immune response [29]. The present study demonstrated that SophorOx™ inhibited and reduced the levels of proinflammatory cytokines, namely, IL-1*β* (50%), IL-6 (75%), and CRP (50%) in exercised SD rats at the terminal phase of exposure. At the same time, the levels of TNF-*α* remained unchanged across the groups. This result is allied with a similar study by Parveen et al. [30], which reported that a combination of herbal extracts (quercetin and rutin) reversed the increased cytokine levels in cyclophosphamide-induced immunosuppressed mice.

Quercetin and rutin are reported to work as antioxidants in stress conditions by fixing Fe^+2^, thus preventing the generation of highly reactive free radicals [31]. Extensive studies have been conducted on quercetin and rutin to improve stress conditions in the rat model, specifically emphasising malondialdehyde levels. In our present trial, we have reported the synergistic effect of Q-R on various oxidative markers (8-iso-PGF2*α*), (MDA), and (8-OHdG) and inflammatory markers (CRP, IL-1*β*, and IL-6). Thus, the trial suggests that SophorOx™ is a potent antioxidant and anti-inflammatory agent with a prominent ability to block oxidative stress and inflammation mediators. In a future perspective, exercise models can be planned to examine oxidative stress levels in tissues such as skeletal muscle, the various vital organs (lungs, liver, kidneys, and spleen), and serum levels.

## 5. Conclusion

SophorOx™ was screened for its ability to inhibit oxidative stress and reduce the production of proinflammatory cytokines in a treadmill-based rat model. Daily oral administration of SophorOx™ did not adversely affect the animals throughout the experimental period. Exercise caused a significant increase in oxidative stress marker and proinflammatory marker levels. However, oral administration of SophorOx™ in exercised animals significantly decreased oxidative stress and inflammatory biomarker levels. As demonstrated in an exercised rodent model, we conclude that SophorOx™ is a powerful antioxidant and anti-inflammatory agent. SophorOx™ will be clinically tested in humans to evidence its therapeutic properties in the next step.

## Data Availability

No data were used to support the findings of this study.
